# A retrospective comparison of two different immobilization systems for radiotherapy of extremity soft tissue sarcomas and its influence on CTV-PTV margin

**DOI:** 10.1186/s43046-021-00076-2

**Published:** 2021-09-27

**Authors:** Reham Mohamed, Muhammad Shuja, Asaad Al-Hazienh, Moamen Aly

**Affiliations:** 1 Radiotherapy and Nuclear Medicine Department, Cairo University, National Cancer Institute, Cairo, Egypt; 2 King Fahad Medical City, Radiation Oncology Department, Riyadh, Saudi Arabia; 3 Radiation Oncology Department, Mayo Clinic, Rochester, USA; 4 King Fahad Medical City, Radiation Oncology Department, Radiation therapy unit, Riyadh, Saudi Arabia; 5 King Fahad Medical City, Medical Physics Department, Riyadh, Saudi Arabia; 6 South Egypt Cancer Institute, Medical Physics Unit, Radiotherapy and Nuclear Medicine Department, Assiut University, Assiut, Egypt

**Keywords:** Sarcoma, Extremity, PTV

## Abstract

**Background:**

On account of extremity wide range of movements and difficulty of reproducibility during irradiation of extremity sarcomas, assorted immobilization strategies are employed to eliminate setup errors. The study purpose was to compare the setup errors of the commonly used immobilization tools and to define planning target volume (PTV) margins for each device.

**Methods:**

A retrospective review comparing Vac-Loc™ and thermoplastic cast (Tcast) was conducted. On radiotherapy treatment, portal imaging was matched with the pre-treatment simulation imaging for both fixation tools. The isocenter shifts and total vector error (TVE) were compared. Random (σ) and systemic errors (Σ) were computed and PTV margins were defined.

**Results:**

Three hundred seven shifts in each direction measured in 14 patients. Mean displacements for the Vac-Loc™ and Tcast, respectively, were as follow: vertical; -0.01 cm vs. 0.02 cm, longitudinal; 0.03 cm vs. 0.04; lateral; 0.04 cm vs. 0.00 cm and TVE; 0.15 cm vs. 0.17 cm with no significant statistical difference. Random and systemic errors were comparable for both devices. The lateral displacement and rotational random errors were higher Vac-Loc™ compared to Tcast. Overall measured PTV margins were marginally lower for Tcast compared to Vac-Loc™.

**Conclusion:**

Vac-Loc™ and Tcast are valid options for immobilization with no clear superiority of either device. The marginal advantage of Tcast warrants further prospective studies.

## Background

The quality of life for patients with extremity soft tissue sarcomas (STSs) is markedly improved by accurate delivery of radiation, lower radiation dose and introducing the concept of preoperative radiation vs postoperative radiation [1–3]. The radiotherapy (RT) for extremities is challenging for radiation oncologists as setup errors are inherent in the process RT and more prominent for such cases [4].

There is general agreement that sparing part of the subcutaneous lymphatics is of high value to decrease the extremity lymphedema and subcutaneous fibrosis [5, 6], which is really time taking and laborious process especially in large tumors with bigger PTV. To date, there is no standard tool established for extremities immobilization; the most common techniques used include; the negative pressure vacuum air cushions (Vac-Loc™), alpha cradles and thermoplastic casts. Further there is no consensus for PTV margins around clinical target volumes (CTV); however, most of the centers agree upon 0.5–1.5 cm clinical target volume to planning target volume (CTV-PTV) margins [7, 8], which mainly depends on the fixation methods and availability of image guided radiation therapy (IGRT). RTOG 0630 protocol used 5 mm CTV to the PTV margins using daily IGRT for STSs of extremities. The daily IGRT is a good choice for reduction of PTV margins as a general radiotherapy principal. However, the high probability of limbs rotations and diversity of joint movements warrants the use of proper fixation methods in addition to IGRT to achieve least CTV-PTV margins.

In Our department we are using mainly the Vac-Loc™ and thermoplastic casts as immobilization devices for treating STSs of extremities.

We aimed to compare the differences between the two methods in terms of the setup errors and appropriate CTV-PTV margins required for each method.

## Methods

A retrospective study was conducted to review patients with STSs of extremities treated with radiation therapy using Vac-Loc™ and thermoplastic mask as immobilization devices. The Institutional Review Board (IRB) approved the study before starting data collection and all research steps were performed in accordance with relevant guidelines and regulations. Only, patients who gave Informed consent for treatment purpose either personally or by their legal guardians were included in the study.

**Fig. 1:**
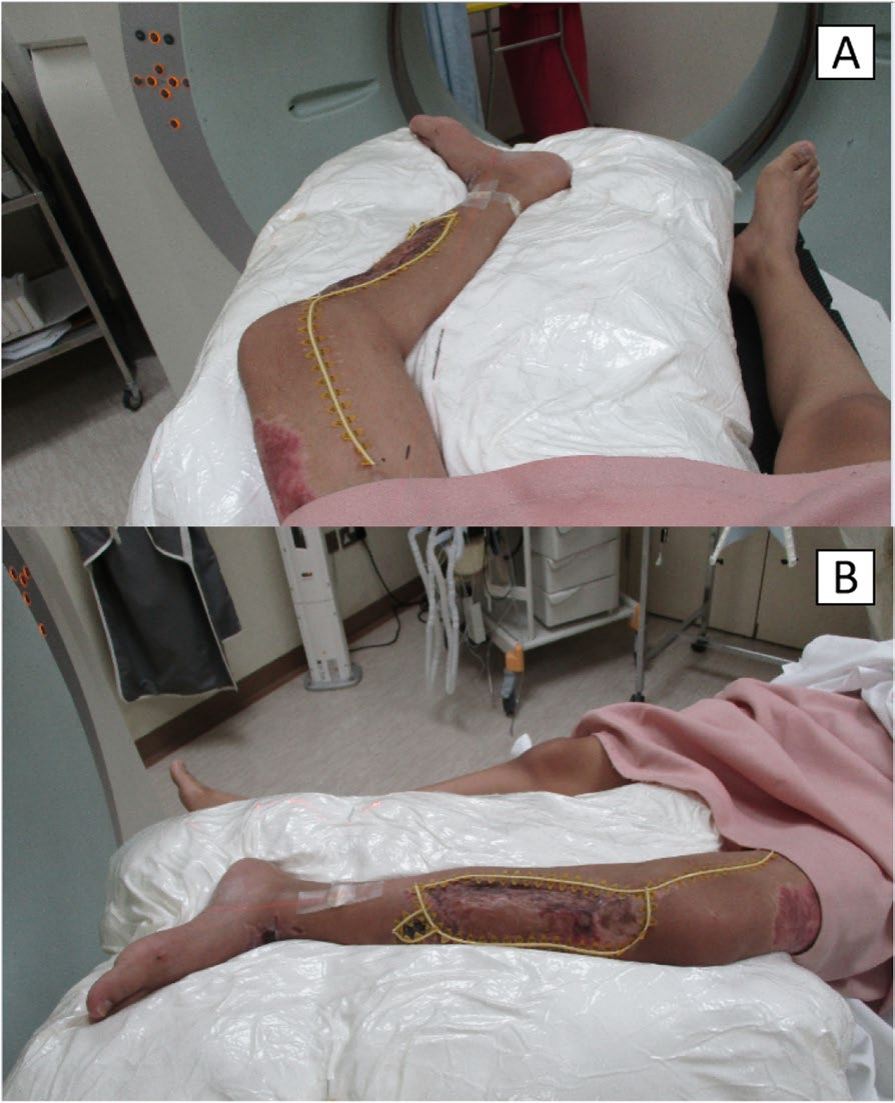
Patient with Vac-Loc immobilization device. The figure showed Vac-Loc ™ immobilization device with landmarks on the patient and cushion (**A**  anterior view and **B** lateral views)

**Fig. 2:**
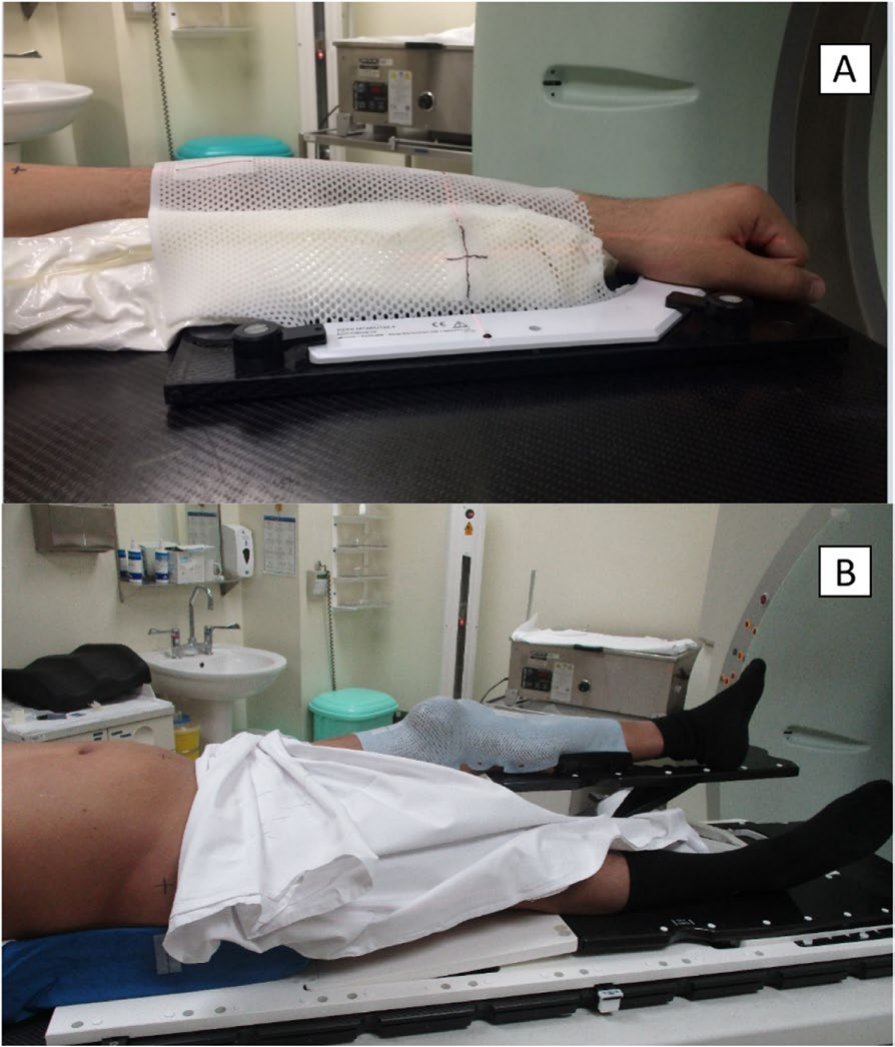
Patients with thermoplastic cast immobilization device. The figure showed thermoplastic cast immobilization device for 2 patients (**A** patient with upper extremity soft tissue sarcoma and **B** patient with lower extremity soft tissue sarcoma) with landmarks on the patient and the cast

**Fig. 3:**
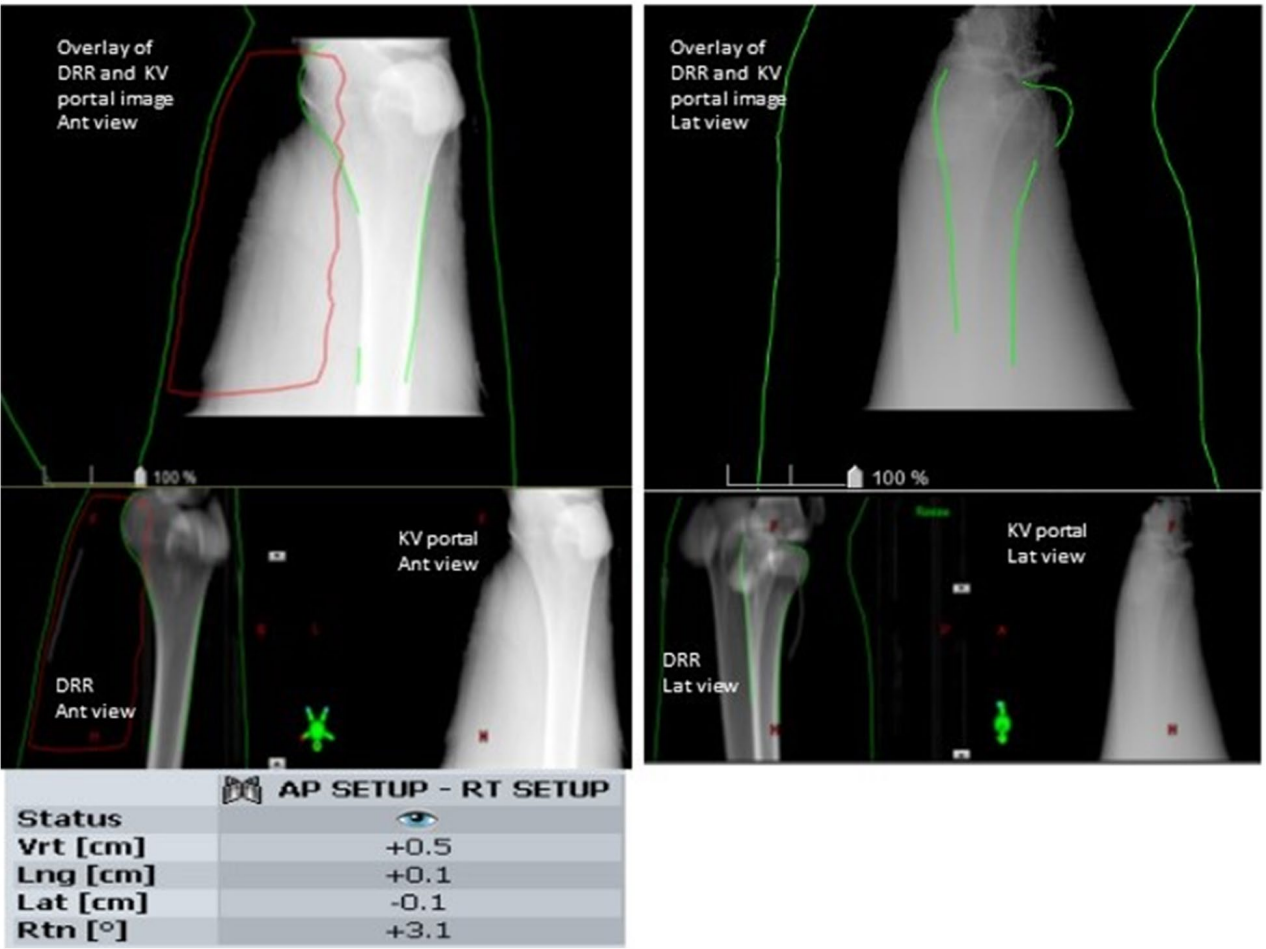
Online KV portal image matching with DRR for a case of Vac-Loc™ fixation. The figure shows the overlay of the digitally reconstructed image (DRR) of CT simulation and the KV portal image taken during treatment for a patient immobilized using the Vac-Loc™ in the anterior and lateral views. Bony structure and body contour delineated on the DRR by green color was matched with KV portal images. The red color represents the PTV. The computerized system generated table showed the isocenter shifts measured in cm in Vrt (vertical), Lng (longitudinal), Lat (lateral) and Rtn (Rotational) directions for that patient on the treatment day

The departmental policy and procedures for STSs of extremities radiotherapy include:

- 1- Patient Positioning and Computed tomography (CT) simulation
  The radiation therapists reviewed simulation form and informed consent obtained from the patient as per the regulations before starting the simulation process. A comfortable reproducible position was chosen and immobilization method was applied based on the recommendations of primary consultant and discussion with the -CT simulation- radiation therapist. In case of Vac-Loc, the iso-center was marked by skin tattoos (anterior and lateral marks) and adding extra-marks on Vac-Loc^TM^ on the same extension of isocenter (Fig.1).
  In case of thermoplastic mask, after molding the U-shaped cast, isocenter was marked on the mask itself with extension of the longitudinal laser marks over the skin area outside U frame and applying skin tattoo as extra-marks (Fig.2). Then CT slices of thickness of 5 mm were obtained and were transferred to the ECLIPSE treatment planning system.
- 2- Target volume, organs at risk (OAR) delineation and treatment planning:
  *For preoperative radiotherapy (RT),* gross tumor volume (GTV) was delineated including the tumor and peri-tumoral edema visible on magnetic resonance imaging (MRI). *For post-operative RT*, the surgical bed, the tumor area, scar and drain sites were delineated. Respecting the anatomical barrier, a longitudinal margin of 2-3 cm and radial margin 1-1.5 cm based on tumor grade and size were added to create CTV [9]. PTV is created by adding 10 mm around CTV. The prescribed dose is 50 Gy in 25 fractions for Preoperative RT and 60-66Gy in 30-33 fractions for post-operative RT using either 3-Dimensional conformal RT (3DCRT) or intensity-modulated radiation therapy (IMRT).
- 3- RT treatment verification
  All patients were treated using the same set-up as for CT-simulation. On Board imaging (OBI) was obtained using orthogonal Kilo voltage (KV) portal imager on the first three consecutive days of treatment, and then weekly for each patient. Bony landmarks were used for matching between the on treatment KV portal imaging with the CT simulation created digitally reconstructed radiographs (DRRs) (Fig 3). This included four directions: vertical, longitudinal, lateral and rotational.

### Estimation of Reproducibility

The angular and translational displacements were calculated by experienced radiation therapists. The 1^st^ image taken each day was taken into consideration for comparison and not the online corrected image (in case of IGRT). The translational set up error includes the vertical, longitudinal and lateral directions and measured in centimeters. To evaluate the overall shift from isocenter, TVE was calculated which is a mathematical function that takes (x, y and z) shifts into account simultaneously. TVE for each patient ($i$) was computed from the mean displacement ($m$) in the three directions: ${\boldsymbol{T}}{\boldsymbol{V}}{\boldsymbol{E}}=\surd {{\boldsymbol{m}}}_{{\boldsymbol{x}}}^{2}+{{\boldsymbol{m}}}_{{\boldsymbol{y}}}^{2}+{{\boldsymbol{m}}}_{{\boldsymbol{z}}}^{2}$

As TVE did not count rotational (angular) displacements, it is calculated separately and measured in angular degrees. Systematic (Σ) and random (σ) errors for the studied population were calculated according to Stroom and Heijmen [10].

The systematic error (Σ) for each immobilization device is the standard deviation (SD) of the mean ($m$) in each direction for each patient: ${\boldsymbol{\Sigma }}_{{\boldsymbol{x}}}={\boldsymbol{S}}{\boldsymbol{D}}\left({{\boldsymbol{m}}}_{{\boldsymbol{i}},{\boldsymbol{x}}}\right)$**.**

While, the random error (σ) for each immobilization device is the square root of the squared displacement standard deviation: ${{\boldsymbol{\sigma}}}_{{\boldsymbol{x}}}=\surd {{\boldsymbol{S}}{\boldsymbol{D}}}_{{\boldsymbol{i}},\boldsymbol{ }{\boldsymbol{x}}}^{2}$

To calculate the minimum required PTV margin around the CTV, we utilized the van Herks’ formula to ensure a minimum dose of 95% to the clinical target volume for 90% of the fractions, i.e. allowing significant dose discrepancies in around 10% of sessions [11].

$${\boldsymbol{M}}{\boldsymbol{a}}{\boldsymbol{r}}{\boldsymbol{g}}{\boldsymbol{i}}{\boldsymbol{n}}\boldsymbol{ }=\boldsymbol{ }2.5\boldsymbol{ }\boldsymbol{\Sigma }\boldsymbol{ }+\boldsymbol{ }0.7{\boldsymbol{\sigma}}$$

### Statistical analysis

The translational and angular mean displacement were compared for Vac-Loc™ and thermoplastic cast. Shapiro Wilkes and Levene’s tests were used to calculate normal distribution of data and equal variances respectively. *P* value < 0.05 was considered significant. Two-tailed independent sample t-test was performed to compare the data between the 2 cohorts. The random error and systematic errors were measured and then, PTV margins were calculated using the Van Herks’ formula for both cohorts.

## Results

Twenty-three patients with STSs of extremities were treated during the defined study period at our department. We excluded nine patients because they are treated by different protocols and did not fulfill study eligibility criteria. Fourteen patients were eligible for analysis; 7 patients treated with Vac-Loc™ and 7 patients with thermoplastic cast.

### Patient characteristics

Patients’ characteristics are shown in Table 1. Median age of patients was 43.8 years (range: 18–74). Six patients (42.9%) were females, and 8 (57.1%) were males. Predominant site was lower extremity (57.1%) and common histopathology was liposarcoma (42.9%). Mean tumor size was 8.8 cm (range: 2.5–18 cm). Preoperative RT was given to 5 patients (35.7%) and postoperative RT was given to 9 patients (64.3%).

**Table 1:** Patient characteristics

Variable	Vac-Loc ™	Thermoplastic cast	All patients
Number	7	7	14
Age (YEARS)	41(18–73)	46.7(25–74)	43.8(18–74)
Gender			
Male	3 (42.9%)	3 (42.9%)	6(42.9%)
Female	4 (57.1%)	4 (57.1%)	8(57.1%)
Extremity			
Upper	5 (71.4%)	1 (14.3%)	6(42.9%)
Lower	2 (28.6%)	6 (85.7%)	8(57.1%)
Histopathology			
Liposarcoma	3 (42.9%)	2 (28.6%)	5 (35.7%)
Fibromatosis	2 (28.6%)	1 (14.3%)	3 (21.4%)
Synovial	2 (28.6%)	-	2 (14.3%)
Sarcoma	-	2 (28.6%)	2 (14.3%)
Undifferentiated			
Sarcoma	-	2 (28.6%)	2 (14.3%)
Others			
Size (CM)	7 (3.2–15)	9.8 (2.5–18)	8.8 (2.5–18)
Laterality			
Right	2 (28.6%)	3 (42.9%)	5 (35.7%)
Left	5 (71.4%)	4 (57.1%)	9 (64.3%)
Radiotherapy			
Preoperative	2 (28.6%)	3 (42.9%)	5 (35.7%)
Postoperative	5 (71.4%)	4 (57.1%)	9 (64.3%)

### Setup errors and PTV margin calculation

**Fig. 4:**
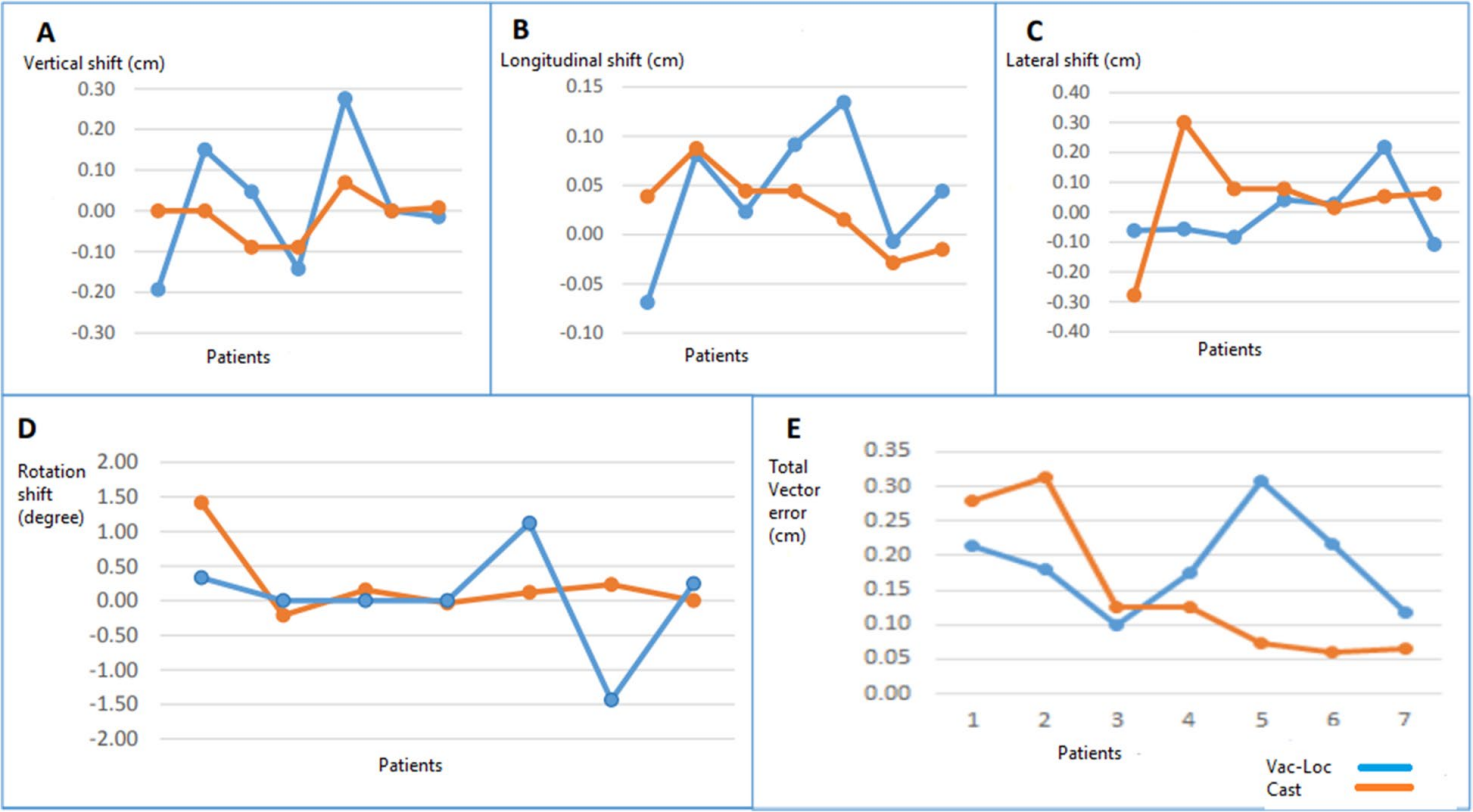
Comparison of Vac-Loc™ and thermoplastic cast; Translational and angular shifts. The figure shows comparison of the mean translational, angular and Total Vector Error (TVE) displacements for each patient immobilized using Vac-Loc™ and thermoplastic cast. The mean value for each patient in **A**  Vertical direction **B** Longitudinal direction **C** Lateral direction **D**  Rotational displacement and **E** TVE is measured from all portal images taken to that patient during the whole course of treatment

In total, 307 measurements of set-up errors in each direction were collected from the ARIA offline imaging (average of 22 sets of measurements per patient). Mean displacements (m) for translational, angular and Total Vector Error (TVE) for customized Vac-Loc™ immobilizer (Group 1) and thermoplastic cast (Group 2) are shown in Table 2 and Fig. 4.

**Table 2:** Mean translational and angular shifts Comparison of Vac-Loc™ and thermoplastic cast

*Variable*	*Vertical (cm)*	*P value*	*Longitudinal (cm)*	*P value*	*Lateral (cm)*	*P value*	*Rotational (Degree º)*	*P value*	*TVE*^*a*^* (cm)*	*P value*
*Vac-Loc™*	-0.01 ± 0.16	0.63	0.03 ± 0.07	0.6	0.04 ± 0.17	0.55	0.04 ± 0.73	0.58	0.15 ± 0.10	0.44
*Thermoplastic cast*	0.02 ± 0.06		0.04 ± 0.04		0.00 ± 0.11		0.24 ± 0.54		0.17 ± 0.07	

^a^ TVE (total vector error)

The vertical setup error was -0.01 ± 0.16 cm for group 1 vs. 0.02 ± 0.06 cm for group 2 (*p* value = 0.63). The longitudinal setup error was 0.03 ± 0.07 cm *vs* 0.04 ± 0.04 cm (*p* value = 0.60) and lateral displacement was 0.04 ± 0.17 cm vs. 0.00 ± 0.11 cm (*p* value = 0.55) for group 1 and 2 respectively. It was also noticed that there was no statistical difference between thermoplastic cast and Vac-Loc™ regarding the mean translational displacement. The total vector error was also not statistically different in both immobilization methods; 0.15 ± 0.10 for Vac-Loc ™ vs. 0.17 ± 0.07 for thermoplastic cast (*p* value = 0.44).

The rotational displacement was more (0.24 ± 0.73) in the Vac-Loc™ compared to 0.04 ± 0.54 in thermoplastic cast but was also statistically insignificant *p* value 0.58.

The systematic errors for Vac-Loc™ were 0.16 cm, 0.07 cm, 0.17 cm in the vertical, longitudinal and lateral directions respectively compared to 0.06 cm, 0.04 cm, 0.11 cm for thermoplastic cast patients. The lateral displacement random error showed clear difference and was 1.23 cm and 0.56 cm for Vac-Loc™ and thermoplastic cast respectively. The random errors for the vertical and longitudinal directions were 0.59 cm, 0.65 cm for Vac-Loc™ compared to 0.81 cm, 0.56 cm for thermoplastic cast respectively. The rotational random error proved to be higher in Vac-Loc™ compared to thermoplastic cast (3.17º vs 2.26º respectively) and the same for systematic rotational error (0.76º for Vac-Loc™ compared to 0.54º for thermoplastic cast).

The data mentioned above reflected upon CTV-PTV margin. The PTV margins required for Vac-Loc™ were 0.82 cm vertically, 0.62 cm longitudinally and 1.29 cm in lateral direction. In Comparison, the margins required for thermoplastic mask were lesser (0.71 cm, 0.49 cm and 0.67 cm in the vertical, longitudinal and lateral directions respectively). Applying Van Herks’ formula, Table 3 showed approximately CTV-PTV margins of 1.0—1.5 cm would be required for Vac-Loc™, which is substantially larger than the 1.0 cm margin routinely used in our department. On the other hand, approximately 0.5 to 1 cm is enough CTV-PTV margin in thermoplastic cast cases.

**Table 3:** Random/ Systematic error and PTV margins for each immobilization technique

Variable	*Random error*				*Systemic error*				*PTV Margin (Van Herk’s)*		
	**Vertical**	**Longitudinal**	**Lateral**	**Angle**	**Vertical**	**Longitudinal**	**Lateral**	**Angle**	**Vertical**	**Longitudinal**	**Lateral**
**Vac-Loc™**	0.59	0.65	1.23	3.17	0.16	0.07	0.17	0.76	0.82	0.62	1.29
**Thermoplastic Cast**	0.81	0.56	0.56	2.26	0.06	0.04	0.11	0.54	0.71	0.49	0.67

Random errors, systemic errors and PTV margins measured in cm. Angular rotation random and systemic errors measured in Degree

The table shows the random and systematic errors and the PTV margins required for both Vac-Loc™ and thermoplastic cast.

## Discussion

Comparison of radiotherapy setup errors among previously published studies for extremities STSs, should be taken cautiously. The practiced immobilization technique logically affects the daily setup and PTV margins required. The manufacturers of each immobilization device have their own recommendations regarding the material molding and retraction [1, 4]. To our knowledge there is limited data comparing setup errors for different immobilization devices used for extremities RT. Since, daily IGRT was not routinely used in our department; running this study is justified for determination of CTV-PTV margins.

The data retrieved from our study showed the mean translational displacements in Vac-Loc™ patient -0.1 mm, 0.3 mm, 0.4 mm vertically, longitudinally and laterally respectively, and the mean translational displacements of thermoplastic mask were 0.2 mm, 0.4 mm and 0 mm in the vertical, longitudinal and lateral direction respectively. There was no significant statistical difference between the two group of patients. However, a marginal trend of larger discrepancy among our patients treated with Vac-Loc™ was noticed. The possible reasons for comparable accuracy and reproducibility could be related to the extra set-up marks drawn by our therapists.

These set up errors were lower compared to data of Princess Margret Hospital (PMH) retrospective study by Kim et al., directed mainly to STSs of upper extremity [12]. A total of 17 patients were compared for immobilization; vacuum cradle with thermoplastic shell, vacuum cradle, and none. Mean translational displacements in the vertical, longitudinal and lateral directions were 3.2 ± 3.3 mm, 1.2 ± 2.5 mm and -0.4 ± 3.3 mm respectively for vacuum cradle with thermoplastic shell. The vacuum cradle vertical, longitudinal and lateral mean displacements were 0 ± 1.7 mm, -0.7 ± 2.2 mm, and 2.5 ± 1.0 mm. The total vector error was not measured in this study. There were significant differences in systematic error values for all translational and rotational axes between immobilization methods at PMH data in contrast to non- significant difference in our data (TVE 1.5 ± 1.0 mm for Vac-Loc™ vs 1.7 ± 0.7 mm for thermoplastic cast). However, in our study had different type of patients, with 60% of our cohort was with STSs lower limb while all patients of PMH are upper limb. Practically, the upper limb sarcoma is more challenging compared to lower limb sarcoma regarding reproducibility of setup.

The other important study showed comparable results to our data is the RTOG 0630 that enrolled 98 patients from 18 institutions [13]. Different fixation tools were used with IGRT; 45 patients were verified by KV imaging, as in our study. RTOG 0630 used 0.5 cm as CTV-PTV margin. The translational shifts reported in the 45 patients were − 0.5 ± 4.0 mm, 0.0 ± 2.3 mm and − 0.5 ± 3.2 mm in the lateral, longitudinal and vertical displacements respectively. These data from RTOG was in agreement with our study however; RTOG reported day-to-day set up errors of up to 20 mm in one or more directions in absence of IGRT [14].

CTV-to-PTV margins estimated in RTOG 0630 study is of great importance as it was measured for statistically sufficient number of patients using KV portal imaging and cone beam CT (CBCT). The RTOG used 15 mm for CTV-PTV margin if daily IGRT was not used, which was way larger than the 5 mm margin used in the protocol of daily IGRT. The impact of reduced margin on the toxicity was published by the same group with proven reduction of the late toxicity (15). Our study on the other hand set the margins of approximate 1 cm to 1.5 cm for Vac-Loc™ (0.82 cm vertically, 0.62 cm longitudinally and 1.29 cm in lateral direction) and approximate 0.5 to 1 cm for thermoplastic cast (0.71 cm, 0.49 cm and 0.67 cm in the vertical, longitudinal and lateral directions respectively).

The rotational displacement in our patients was 0.24 ± 0.73 in the Vac-Loc™ and 0.04 ± 0.54 in thermoplastic. The highest systemic and random rotational errors were in Vac-Loc patients (0.76º and 3.17º respectively). In PMH study, 18% of vacuum cradle with thermoplastic mask and 15% of vacuum cradle alone needed repositioning with rotation more than 5°. The RTOG 0630 found that most of their patients had a rotation less than 1.5° and based on these results, they justified the practice of applying the translational shift by using IGRT without proper correction of the rotational shift prior to that [12, 13].

Apart from 1.5° rotational displacement acceptability of RTOG and the 5° used in PMH, the concept of cut of value 3° angular displacement is popular, and we concur with this in our department. Reference to that our thermoplastic cast data that showed less than 3° rotation compared to the Vac-Loc ™ could by advisable specially in absence of 6-degree freedom couch capable for correction of rotational errors.

Limitations to our study were; (a) relatively smaller sample size, (b) we evaluated only interfractional displacement, but not intra-fractional shifts, (c) variation in tumor locations and (d) satisfaction survey for using immobilization devices was not conducted for treating radiation therapists.

## Conclusion

There is a notable scarcity of evidence regarding the optimal immobilization devices for STSs of extremities. We found that both Vac-Lok™ and thermoplastic casts were reliable, accurate and rendered highly accurate reproducibility.

Our data should be analyzed cautiously due to small number of patients, however most of the relevant studies had similar limiting factor. The results of the present study warrant multi-institutional randomized trial including larger number of patients to determine both inter- and intrafraction errors that would help to identify the optimal immobilization devices for use in STSs of extremities.

## Data Availability

Research data are stored in our institutional repository and will be shared upon request to the corresponding author.

## Abbreviations

STSs: Soft tissue sarcomas
PTV: Planning Target Volume
Tcast: Thermoplastic cast
TVE: Total vector error
σ: Random error
Σ: Systemic error
CTV: Clinical target volumes
CTV-PTV: Clinical target volume to planning target volume
IGRT: Image guided radiation therapy
IRB: Institutional Review Board
CT: Computed tomography
OAR: Organs at risk
*RT*: *Radiotherapy*
GTV: Gross tumor volume
MRI: Magnetic resonance imaging
3DCRT: 3-Dimensional conformal radiotherapy
IMRT: Intensity-modulated radiation therapy
OBI: On Board imaging
KV: Kilo voltage
DRRs: Digitally reconstructed radiographs
SD: Standard deviation
m: Mean
PMH: Princess Margret Hospital
CBCT: Cone beam computed tomography
